# *Operando* tracking of ion kinetics and state-of-charge via multiresonant fiber-optic grating sensors in sodium-ion batteries

**DOI:** 10.1038/s41377-026-02388-1

**Published:** 2026-06-26

**Authors:** Xile Han, Jinliang Li, Wen Wu, Jiajian Long, Xumiao Chen, Yongqi Li, Man Chen, Gaozhi Xiao, Yongjin Fang, Wenjie Mai, Tuan Guo

**Affiliations:** 1 Institute of Photonics Technology, College of Physics & Optoelectronic Engineering, Jinan University, Guangzhou, 510632 China; 2 Department of Physics, College of Physics & Optoelectronic Engineering, Jinan University, Guangzhou, 510632 China; 3 College of Chemistry and Molecular Sciences, Hubei Key Laboratory of Electrochemical Power Sources, Wuhan University, Wuhan, 430072 China; 4 China Southern Power Grid Power Generation Company Limited, Energy Storage Research Institute, Guangzhou, 510080 China; 5 Advanced Electronics and Photonics Research Centre, National Research Council of Canada, Ottawa, K1A 0R6 Canada

**Keywords:** Optical sensors, Fibre optics and optical communications

## Abstract

Ion transport within the sub-micron diffusion layer at the electrode–electrolyte interface governs battery function, yet probing its rapid, confined dynamics under operating conditions remains a challenge. Here, we introduce a compact, highly sensitive multiresonant fiber-optic grating sensor that monitors these processes operando in sodium-ion batteries without interfering with their operation. Using this approach, we reveal an intermediate stage of ion transport between adsorption and diffusion at the interface. We find that a shorter duration of this intermediate stage correlates with superior fast-charging performance. Furthermore, by integrating the sensor’s optical intensity in real time, we achieve state-of-charge quantification with unprecedented accuracy (>98%). This operando measurement platform offers a new capability for battery diagnostics and can inform the design of next-generation batteries with enhanced electrochemical properties.

## Abstract (web summary)

Operando monitoring of interfacial ion kinetics and real-time accurate state-of-charge quantification in sodium-ion batteries has been achieved using a compact multiresonant fiber-optic grating sensor.

## Graphical abstract

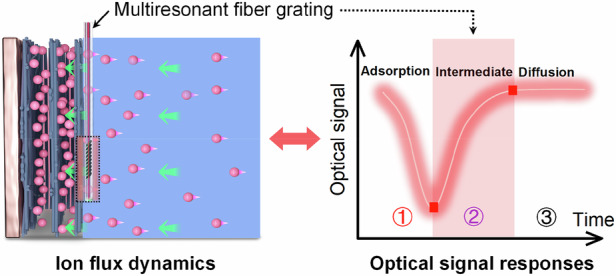

## Introduction

Sodium-ion batteries (SIBs) have garnered significant attention in energy storage research owing to their cost-effectiveness and high energy density^1^. However, to meet the stringent requirements of large-scale energy storage technologies, SIBs need to demonstrate high standards of safety, reliability, and stability^2^. The performance of SIBs is primarily dictated by the (de)sodiation processes, which depend on the dynamics of Na-ion (de)intercalation between electrodes and electrolytes^3^. Understanding the complex electrochemical reactions over repetitive (de)sodiation cycles and decoding their reliance on ion transport dynamics at the electrode-electrolyte interface are still unresolved issues. Current methodologies for the quantification of ion migration near the electrode are largely limited to studies of non-operational batteries or non-operando measurements^4,5^. To overcome these limitations, various in-situ techniques, including transmission electron microscopy^6^, nuclear magnetic resonance^7^, X-ray diffraction (XRD)^8^, and Raman spectroscopy^9^, have been proposed for studying the internal dynamic processes of SIBs. However, the reliance on sophisticated laboratory tools for these in-situ techniques hinders routine monitoring of batteries during in-field applications.

In addition, accurate estimation of state-of-charge (SoC) is another critical challenge in battery development. Conventional methods for SoC estimation often suffer from low precision, leading to operation under suboptimal conditions that can compromise battery health and safety^10–12^. The voltage-SoC relationship in SIBs exhibits pronounced nonlinearity, particularly in low and high SoC regions where voltage fluctuations are minimal, posing substantial challenges to accurate assessment^13,14^. Although advanced estimation techniques, such as Coulomb counting combined with algorithmic modeling, have been employed^15^, those techniques still remain susceptible to inaccuracies induced by temperature fluctuations and aging effects. Thus, developing innovative methodologies that offer a deeper understanding of the kinetic behavior of SIBs and enable precise SoC monitoring in-real time is imperative for advancing their practical deployment.

To tackle these challenges, it is essential to develop new technologies based on implanted sensors compatible with harsh electrolyte environments^16–18^. Given the chemical stability of silica glass, the utilization of optical detection technology based on silica glass emerges as a promising choice for developing in-situ sensors^19,20^. Due to their high sensitivity, rapid response time, and immunity to electromagnetic interference, optical detection technologies have been extensively developed for characterizing internal parameters^21,22^. Compared to optical detection using bulk optical components, optical fiber sensors offer a promising approach for implantable and operando measurements of the electrochemical behaviors in batteries^23,24^. For example, Yamanaka demonstrated the feasibility of in-situ ion concentration measurements near the electrode surface in lithium-ion batteries by Raman spectroscopy using very thin multi-fiber sensors^25^. More recently, Euser et al. ^26^ and Tarascon et al. ^27^ demonstrated in-operando analyses of electrolyte composition species and the interpretation of electrochemistry using Raman spectroscopy and mid-infrared spectroscopy, respectively. All the above studies demonstrate the capabilities of operando analysis to guide the design of better batteries with improved electrochemical performance. However, current research on optical fiber sensors in batteries has mainly focused on static or quasi-static electrochemistry, leaving the transient states of electrochemical processes at the nanoscale largely unexplored. Preliminary results indicate that modifying the grating of optical fiber grating sensors enables precise measurement of ion concentrations in solutions^28,29^. From a fundamental perspective, Na-ion migration at the electrode-electrolyte interface is closely linked to both ion concentration and migration dynamics^30,31^, offering a promising approach to probe the internal kinetics of SIBs. Notably, the internal ion migration dynamics within SIBs correlate directly with their SoC, suggesting that precise quantification of ion migration could enable SoC estimation at a fundamental physical level^32^. This insight presents a novel strategy for achieving high-precision, quantitative SoC monitoring in SIBs, potentially overcoming the limitations of conventional estimation methods.

**Fig. 1:**
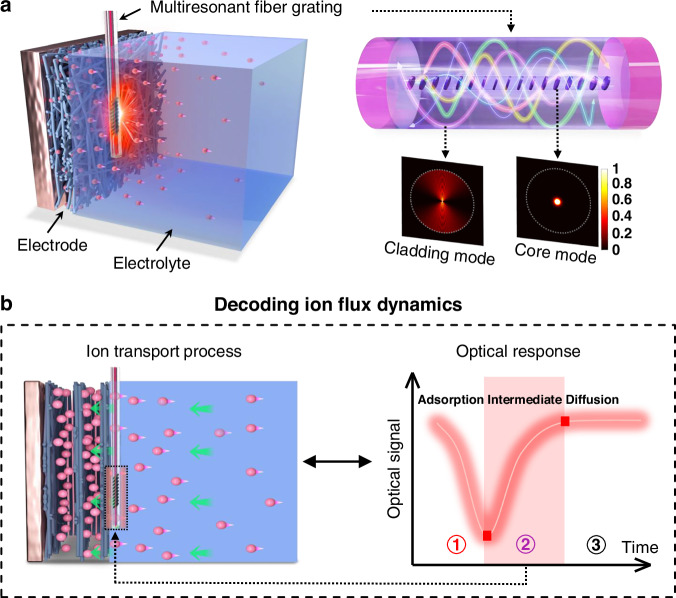
**Schematic of operando decoding of ion transportation dynamics in batteries using advanced optical fiber sensing technology.** **a** Optical fiber sensor capable of being inserted near the electrode surface of a working battery to monitor its ion transportation dynamics without disturbing its operation. The right configuration shows the MFG in the fiber core, which is able to couple light from fiber core to cladding and monitor the surrounding electrolyte concentration and the interface ion transport with high sensitivity. **b** Correlation between the ion dynamic activities and the corresponding optical signals obtained from the optical fiber sensor

Based on this insight, we develop a compact fiber-optic electrochemical sensor that can be implanted directly into a sodium-ion battery near the electrode surface (Fig. 1a). The sensor is based on a single multiresonant fiber-optic grating (MFG), which couples light into cladding modes via diffraction, producing tens of narrow spectral resonances tied to an evanescent field that extends beyond the fiber boundary. These narrow resonances are highly sensitive to minute refractive index changes resulting from local ion transport and concentration shifts at the electrode–electrolyte interface of the SIB (Fig. 1a). By tracking the resonant peak intensity of a cut-off mode, which responds strongly to changes in mode confinement and coupling efficiency near the cut-off boundary, we achieve a refractive index resolution on the order of 10⁻⁶ refractive index unit (RIU), a temperature resolution of 0.1 °C, and high temporal (sub-second) and spatial (sub-μm) resolution^33–35^.

Based on results obtained with the MFG sensor, we observed an “intermediate stage” (colored red in Fig. 1b) between the adsorption and diffusion regions during the Na-ion storage process, which has previously been neither predicted nor experimentally demonstrated^36^. Further studies demonstrated that this intermediate stage plays an important role in the behaviors of ion transport dynamics at the electrode-electrolyte interface. Moreover, we discovered a stable and reproducible correlation between the SoC of SIBs and the integrated optical spectral intensity from the proposed MFG sensor. By integrating the optical intensity from the proposed fiber sensor, we have demonstrated a high accuracy SoC quantification method with an accuracy higher than 98% and real-time readout.

## Results

### Operando monitoring of ion transport at the electrode-electrolyte interface

**Fig. 2:**
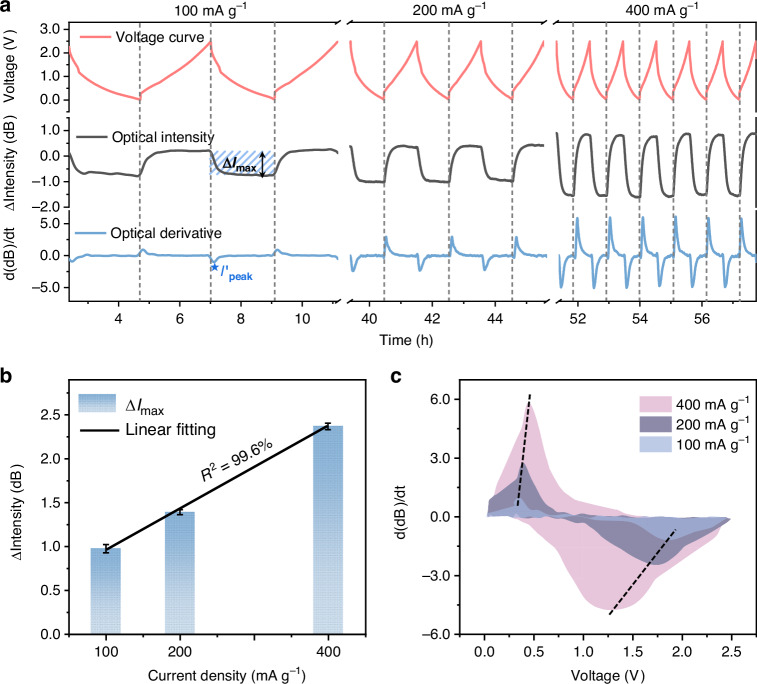
**Operando monitoring of the ion transport.** **a** Real time response of the GCD curve (red), optical intensity (black), and derivative of the optical intensity (blue). **b** Maximum optical intensities of cut-off mode (ΔI_max_), see the blue-shaded area of the optical intensity in Fig. 2a at 100, 200, and 400 mA g^−1^. Each value is obtained by averaging of 5 continuous cycles. **c** Optical intensity derivative d(dB)/dt versus voltage at 100, 200, and 400 mA g^−1^

We used the optical intensity of cladding modes from the MFG sensor to study the interfacial ion transport behavior in SIBs. SnO_2_/BaTiO_3_/C electrodes were employed to validate the method. A photograph of the experimental setup is shown in Fig. S1. Figure 2a displays a galvanostatic charge-discharge (GCD) curve (red), the optical intensity of the cut-off mode (black), and the derivative of optical intensity (blue) to verify the correlation between electrochemical behavior and optical signals in SIBs. We measured ion concentration by recording the intensity of the cut-off mode, which is the mode most sensitive to the surrounding refractive index. The time-resolved intensity change, which is proportional to the refractive index change, correlates with the difference between the bulk electrolyte concentration and the interface ion concentration. We observe a correlation between the GCD curve and the optical intensity, where changes in optical intensity represent refractive index changes of the liquid electrolyte, thus reflecting the changes in ion concentration at the electrode-electrolyte interface. Notably, variations in optical intensity are evident at current densities of 100, 200, and 400 mA g^−1^. We find a stable and repeatable correlation with the corresponding ion transfer rates in the curves. The introduction of the derivative allows us to better capture the Na-ion transport state, both in steady and unsteady conditions, at the electrode interface. The correlation between the GCD curve and the optical intensity over multiple charge-discharge cycles is further verified, as shown in Fig. S2a.

To confirm the relationship of optical intensity and its derivative to current density, we compare the maximum values of optical intensities (ΔI_max_) at 100, 200, and 400 mA g^−1^. The data were obtained by averaging 5 data points, as shown in Fig. 2b. During discharge, the increased flow of Na-ions at higher current densities leads to an increase in ΔI_max_. A thorough investigation of the changes in ΔI_max_ with varying current densities reveals a highly consistent linear relationship, evidenced by a regression coefficient R^2^ of 99.6%. We also compare the relationship between the optical derivative and the voltage at three current densities, 100, 200, and 400 mA g^−1^, as shown in Fig. 2c. To enable comparison, we have shaded the areas of the three closed-loop curves with different fill colors. The larger the loop area, the more intense the dynamics of Na-ion transport at the electrode interface. During discharging, the (negative) peak of d(dB)/dt moves towards the lower voltage with increasing current density. Conversely, during charging, the (positive) peaks shift towards higher voltage with increasing current density. The shifts in peak position directly reflect the evolution rate of Na-ion concentration at different voltages during charging and discharging processes. Similar to the oxidation-reduction current peak in CV curves, the rate of change in Na-ion concentration near the electrode produces peaks at specific voltages. We verified the stability of the relationship between the optical derivative and the electrical potential over multiple cycles, finding that the curves corresponding to the second cycle and beyond almost completely overlap (Fig. S2b).

### Decoupling the ion transport dynamics of adsorption/intermediate/diffusion processes

**Fig. 3:**
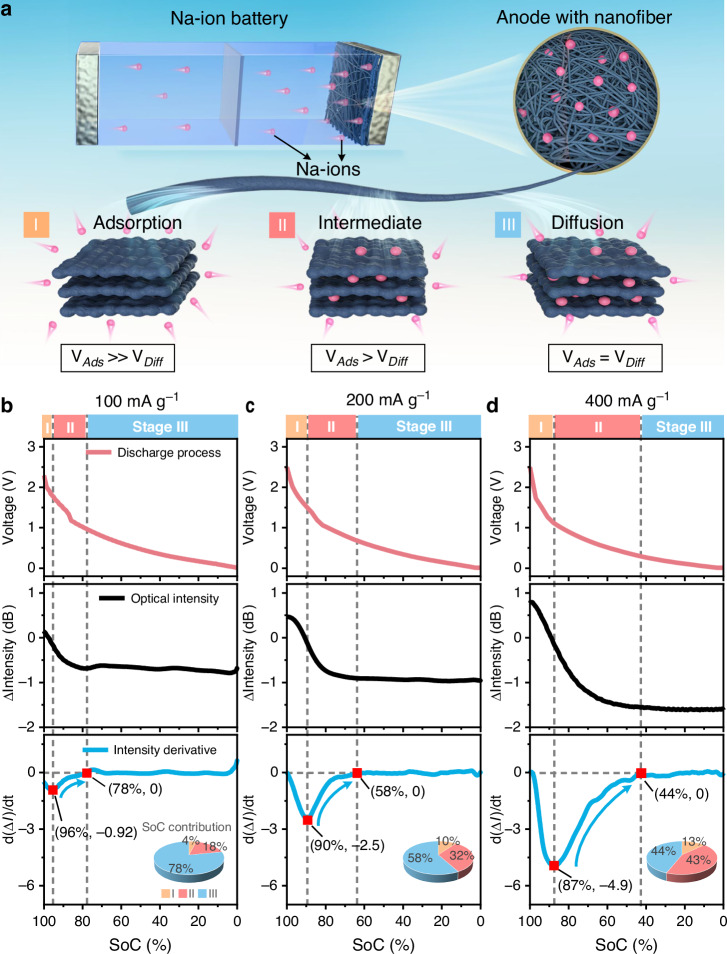
**Operando decoupling of dynamic ion processes during battery operation.** **a** Schematic of adsorption, intermediate, and diffusion states in NIB. Real-time response of GCD curve (red), optical intensity (black), and optical derivative (blue) of NIBs at **b** 100 mA g^−1^, **c** 200 mA g^−1^ and **d** 400 mA g^−1^, in which the colored pie chart insets show the quantitative analysis of adsorption, intermediate, and diffusion states at 100, 200, and 400 mA g^−1^, respectively, based on the optical derivative results

The dynamic behavior of SIBs is demonstrated by adsorption and diffusion processes. Adsorption here refers to the migration of Na-ions from the bulk electrolyte to the electrode interface, where they are subsequently adsorbed^37^. Meanwhile, diffusion pertains to the intercalation/redox reactions of Na-ions at the interface^38^. However, during actual battery operation, the adsorption and diffusion processes often overlap in space. Initially, Na-ion storage in the electrode predominantly occurs through adsorption. As the discharge depth proceeds, an intermediate stage emerges between the adsorption and diffusion processes. Eventually, Na-ion storage transitions into a diffusion stage (Fig. 3a). The precise identification of ΔI_max_, the negative peaks of optical derivatives (I’_peak_), and the capacity contribution of the intermediate stage offers a novel perspective for evaluating the charging behavior of electrode materials. We propose that the location of I’_peak_ at a smaller SoC value indicates superior kinetic performance. When I’_peak_ occurs at 100% SoC, the corresponding current represents the maximum charge-discharge current. Beyond this threshold, although the electrode material can still undergo normal charge-discharge cycles, its reversible capacity will be significantly reduced. Precisely distinguishing between these dynamic states of the electrode is crucial for understanding dynamic phenomena such as fast charging. However, the accurate determination of the range and position of the intermediate stage remains challenging, as there is currently no well-established method to effectively detect this behavior.

To investigate these dynamic states, we conducted studies on corresponding optical signals within the electrolyte. Figure 3b–d illustrates the correlation between the dynamic behavior of SIBs and optical signals during the discharging of the battery. We analyzed discharge curves (red), optical intensity (black), and optical derivatives (blue) at 100, 200 and 400 mA g^−1^. It is evident that during the initial shallow discharge stage, the optical intensity gradually decreases with the decrease of SoC, indicating a rapid rise in Na-ion concentration at the electrode-electrolyte interface under the influence of the electric field^39^. A slope in the decreasing optical intensity reveals a continued increase in Na-ion concentration. This change in optical derivative indicates a change in Na-ion migration rate. As the discharge progresses, the derivative of optical intensities stabilizes, reflecting an equilibrium state of Na-ion concentration between the Na-ions from the bulk electrolyte and those diffusing into the electrode. We observe a stable and reproducible correlation between the optical derivative and the Na-ion migration rate. The negative peaks in the optical derivative (I’_peak_) align with the fastest increase in Na-ion migration rates. Given the linear relationship between optical intensity and refractive index, and consequently with ion concentration, changes in optical intensity indicate variations in Na-ion concentration at the interface of the electrode. Thus, the MFG serves as a Na-ion flux sensor, enabling real-time monitoring of Na-ion quantity during battery operation. During Stage I of discharging, the optical derivative values quickly decrease and achieve the I’_peak_ (Stage I). This behavior indicates the rapid migration of Na-ions from the electrolyte to the interface, corresponding to the adsorption behavior (*v*_*Ads*_»*v*_*Diff*_). With deepening discharge, the optical derivative values gradually increase and return to zero, indicating a gradual increase in Na-ion concentration with the slower Na-ion migration rate. In this process, the ion diffusion behavior at the electrode begins to take place (*v*_*Ads*_>*v*_*Diff*_). However, adsorption continues to dominate as the primary Na-ion storage mechanism for Na-ion storage at the electrode, which we refer to as the intermediate process (Stage II). Eventually, the optical derivative stabilizes at zero, as indicated by the optical intensity reaching its maximum. The peak of optical intensity corresponds to the maximum refractive index, interpreted as an equal number of Na-ions adsorbed at the interface and diffused into the electrode interlayer (*v*_*Ads*_ = *v*_*Diff*_), corresponding to the diffusion process (Stage III).

We also observed that peak optical derivative values, I’_peak_, significantly increased with increasing current densities from 100 mA g^−1^ to 400 mA g^−1^, indicating a positive correlation between Na-ion migration rates and current densities. Furthermore, the SoC at which the derivative reached zero decreased from 78% to 44% with increasing current densities, demonstrating an increased intermediate stage at high current densities. We compared the dynamics of SoC for these three stages and found that at 100 mA g^−1^ (inset in Fig. 3b), the capacity contributions to SoC for Stage I, Stage II, and Stage III were 4%, 18%, and 78%, respectively. When the current density increased to 200 mA g^−1^ (inset in Fig. 3c), the capacity contributions were 10%, 32%, and 58% for Stage I, Stage II, and Stage III, respectively. At 400 mA g^−1^ (inset in Fig. 3d), the capacity contributions were 13%, 43%, and 44% for Stage I, Stage II, and Stage III, respectively. To further confirm the relationship between the charge curves and optical intensity, we provided real-time responses of charge voltage (red), optical intensity (black), and optical derivative of SIBs at different current densities (Fig. S3). After conducting a quantitative analysis, we find that both the electrochemical behavior and optical derivative during charge and discharge processes are reversible. Based on the observed kinetic behavior, we propose that the fast-charging performance of electrode materials can be evaluated by considering the contribution of the intermediate stage. Under the same current density, a smaller intermediate stage indicates better fast-charging performance of the electrode material. Furthermore, when the value of optical derivative (I’) is 0, it reflects an equilibrium between the Na-ion concentration at the electrolyte interface and the Na-ion flux diffusing into the electrode material. When the I’ = 0 position coincides with 100% SoC, we suggest that the electrode material is operating at its maximum current tolerance. This characteristic enables precise determination of the electrode’s limiting current. To further validate the correlation between the intermediate stage and the fast-charging performance of electrode materials, we selected hard carbon anodes, which exhibit inferior kinetic performance, for verification (Fig. S4). The intermediate stage of hard carbon presents contributions of 20%, 40%, and 71% at 100 mA g^−1^, 200 mA g^−1,^ and 400 mA g^−1^, respectively, which consistently surpass those of SnO_2_/BaTiO_3_/C nanofiber electrodes across different current densities, demonstrating that SnO_2_/BaTiO_3_/C nanofibers possess better fast-charging capabilities than hard carbon.

### Operando and real-time quantification of SoC

To access richer electrochemical information, we integrated the optical intensity signal from the fiber sensor. This approach is grounded in the integral form of the transport equation^40^:

$${Q}_{i}=\int \Delta {c}_{i}{dt}$$

Where *Q*_*i*_ represents the total quantity of Na-ions transported through the sensing volume, defined by the grating length and the sub-micron evanescent field extending from the fiber surface, ensuring detection within the diffusion layer. Here, *Δc*_i_ corresponds to the change in Na-ion concentration, which we derive from the sensor’s optical intensity variation, as shown in Fig. S5. During battery cycling, Na-ion transfer and accompanying concentration shifts at the electrode–electrolyte interface are tracked in real time via the resonant intensity changes of the MFG sensor. By temporally integrating Δ*c*_i_, we obtain a direct, real-time measure of *Q*_*i*_, thereby establishing the principle for operando SoC quantification using an implanted MFG sensor in operating SIBs.

**Fig. 4:**
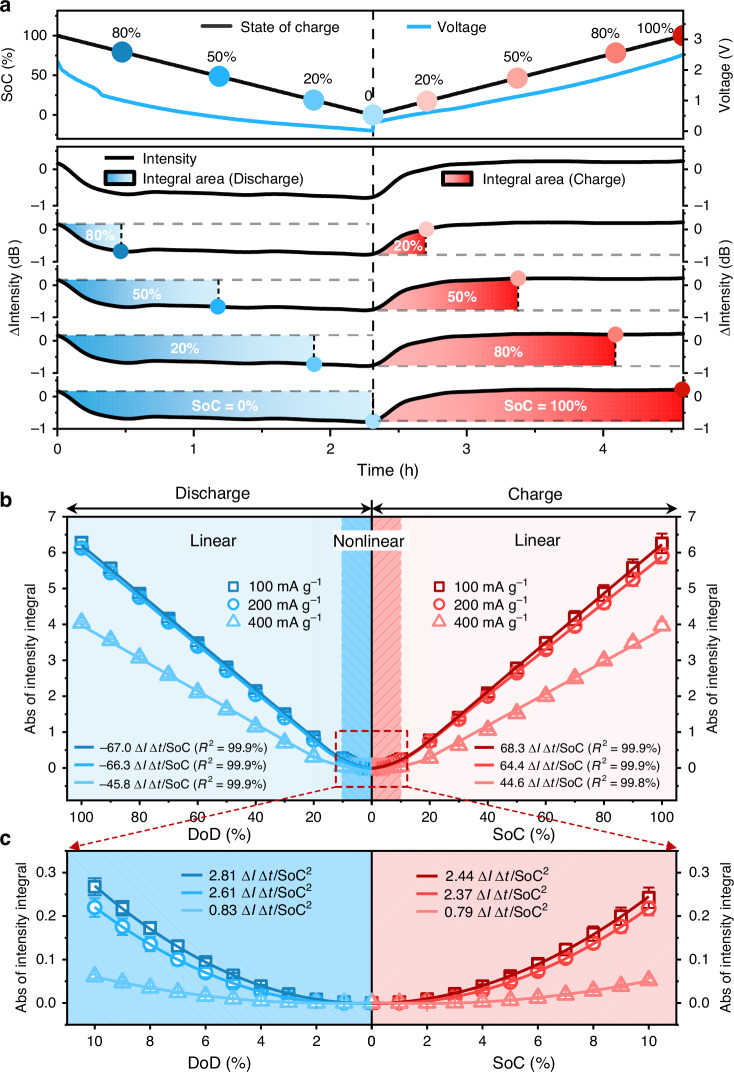
**Operando quantification of SoC.** **a** SoC, GCD curves, and the schematic of 5 integral areas during discharging (blue) and charging (red) as measured by the optical intensities at a current density of 100 mA g^−1^. **b** Relationships between the DoD/SoC and the integral of the change in intensity during discharging (blue) and charging (red). **c** Nonlinear region of Relationships between the DoD/SoC and the integral of the change in intensity during discharging (blue) and charging (red). Each value from **b** and **c** was obtained by averaging over 5 continuous charge and discharge cycles

To validate the applicability of the proposed MFG sensor for monitoring the SoC of SIBs, we integrated the optical intensity corresponding to different depth-of-discharge (DoD) and SoC during both discharge and charge processes, as shown in Fig. 4a. The detailed integration process at varying SoC levels under three different current densities is depicted in Fig. 4b, in which closer points were chosen for the DoD/SoC<10% region. Notably, we found that a highly linear relationship exists between the DoD/SoC and the integrated optical intensity when DoD/SoC are greater than 10%, across all tested current densities. To further confirm this linearity, the relationship between the integrated optical intensity of the MFG sensor and the SoC was measured across 10 repeated charge/discharge cycles at 10% SoC intervals under different current densities. As anticipated, the integrated optical intensity exhibited excellent linearity with SoC (R^2^ > 99.8%), with all tested accuracies exceeding 98.4%, as illustrated in Fig. 4b. A closer inspection of Fig. 4b reveals an intriguing phenomenon in the DoD/SoC ≤ 10% region, where a nonlinear correlation between the integrated optical intensity and SoC is observed. To analyze this nonlinear region, we magnified the data, as shown in Fig. 4c.

The data exhibit a quadratic relationship between the integrated optical intensity and the SoC/DoD, which we attribute to the initial infiltration of Na-ion-rich electrolyte into the porous electrode material. During the initial discharge state, Na-ions are preferentially adsorbed onto the anode material. However, the Na ions in the macroporous structure and those in the bulk electrolyte still maintain an equilibrium. As the Na-ion concentration in the macropores decreases, high-concentration Na ions from the electrolyte diffuse into the macropore region, giving rise to the observed nonlinear process. By mathematically transforming the optical intensity in this nonlinear region, we found that applying a square-root transformation to the integrated optical intensity yielded a linear relationship with DoD/SoC (Fig. S6), further indicating that integrated intensity is proportional to (DoD/SoC) squared with high linearity. Based on the fitting results, the linearity (R^2^ > 99.5%) and accuracy (> 98.4%) remained consistently high across all tested conditions.

Table 1 quantifies the detailed accuracy and linearity of DoD/SoC measurements under various current densities. Interestingly, while both accuracy and linearity slightly decreased with increasing current density, the results remained robust. This slight degradation can be attributed to the significantly accelerated Na-ion flux at high current densities. Under these conditions, the intercalation and deintercalation of Na ions may lag behind rapid current changes, leading to minor inaccuracies in SoC detection.

**Table 1:** Accuracy and linear regression of quantification DoD/SoC at different current densities

Current density	Charge state	DoD/SoC>10%		DoD/SoC≤10%	
		Accuracy	Linearity for DoD/SoC	Accuracy	Linearity for (DoD/SoC)^2^
100 mA g^−1^	Discharge	99.4%	99.9%	99.7%	99.9%
	Charge	99.3%	99.9%	98.6%	99.9%
200 mA g^−1^	Discharge	99.4%	99.9%	99.9%	99.9%
	Charge	99.5%	99.9%	99.8%	99.7%
400 mA g^−1^	Discharge	98.5%	99.9%	99.9%	99.4%
	Charge	98.4%	99.8%	99.9%	98.1%

To further validate the proposed methodology, we applied the same analysis to hard carbon electrodes. Consistent with the above-mentioned results, we observed a strong linear relationship between the optical intensity and DoD/SoC in the high-value region, whereas a nonlinear (quadratic) relationship emerged at low DoD/SoC levels (Fig. S7). Importantly, their integrated intensities are also proportional to (DoD/SoC) squared, with high linearity in the nonlinear region for hard carbon electrodes, yielding high accuracy and linearity (Fig. S8). In summary, the integrated optical intensity scales accurately with the square of (DoD/SoC), which substantially simplifies the subsequent analysis.

## Discussion

The consistent relationship between the real-time optical responses and the charge states of energy storage devices in situ is instrumental in comprehending and assessing the operational performance parameters of SIBs during active service. Optical fiber sensing has emerged as a promising avenue for battery operando monitoring, offering unparalleled advantages such as high sensitivity, real-time monitoring, and non-invasiveness. These compact optical fiber sensors enable their insertion into various hard-to-reach environments for in situ detection, functioning either as a portable probe or as a series of remotely operated devices along a fiber–optic cable. This capability is particularly useful for monitoring batteries in automobiles, domestic installations, and energy storage in power stations.

Decoding physical-chemical-electrochemical parameters of the electrode-electrolyte interface at the nanoscale remains a challenge for researchers. On the basis of the current temperature, strain, and pressure detection, it is also necessary to envisage the implementation of integrated fiber-optic sensors that can simultaneously reveal key electrochemical parameters such as electrolyte aging, dendrite growth, internal gas evolution, SOC, SOH, etc. By combining distributed sensing with the powerful capabilities of machine learning and data fusion algorithms, researchers can extract profound insights from vast datasets and decipher intricate patterns associated with critical battery conditions or failure modes^41^. These analytical strategies will empower predictive maintenance approaches, anomaly detection, and the formulation of robust early warning systems.

Finally, compatibility with existing battery management systems (BMSs) is the key point for practical applications. The deployment of fiber optic sensors needs to take full advantage of their flexibility and small footprints and find ways to embed them directly inside commercial batteries. Unifying real-time optical & electronical monitoring and control technologies within the BMS architecture promises to optimize battery performance, strengthen safety measures, and extend the lifespan of energy storage systems.

## Methods

### Sodium-ion battery

We assembled the SIBs in a glovebox, with the MFG inserted into the battery close to the electrode interface. This configuration facilitates the effective observation of the experimental process. Trimming the free end of the optical fiber eliminates the influence of corresponding strain and polarization cross-sensitivity, ensuring that the spectral response of the MFG is solely attributed to changes in ion concentration at the interface. The electrode material of the SIB is SnO_2_/BaTiO_3_/C nanofibers obtained by electrospinning, which fully utilizes the volume expansion characteristics of alloy materials and the piezoelectric properties of piezoelectric materials to form a micro electric field that promotes Na-ion transport^42^. The scanning electron microscope (SEM) images with element mapping of the SnO_2_/BaTiO_3_/C nanofibers are shown in Fig. S9a–h, which shows the nanofiber structure indicating a uniform spatial distribution of the constituent chemical elements. Figure S9i shows the cross-sectional thickness of the electrode, which measures about 100 μm. Further characterizations of the electrode through XRD and XPS spectra are presented in Fig. S10. To verify the electrochemical performance, we conducted CV, cycling, and rate performance tests of SnO_2_/BaTiO_3_/C nanofibers for SIBs, as depicted in Fig. S11, revealing the stable Na-ion storage performance. Consequently, we selected the SnO_2_/BaTiO_3_/C nanofibers as the working electrode for exploring the dynamic behavior in SIBs. Unless otherwise indicated, the electrodes used in the tests are SnO_2_/BaTiO_3_/C nanofibers. To validate our findings, we also tested hard carbon obtained from BTR New Energy for comparison.

### MFG sensors and their optical characteristics

The MFG sensors are inscribed by using well-established phase mask technology and a pulsed excimer laser. The fibers used are commercial single-mode fibers^43–45^. All the details of MFG fabrication are given in the Supporting Information section.

**Fig. 5:**
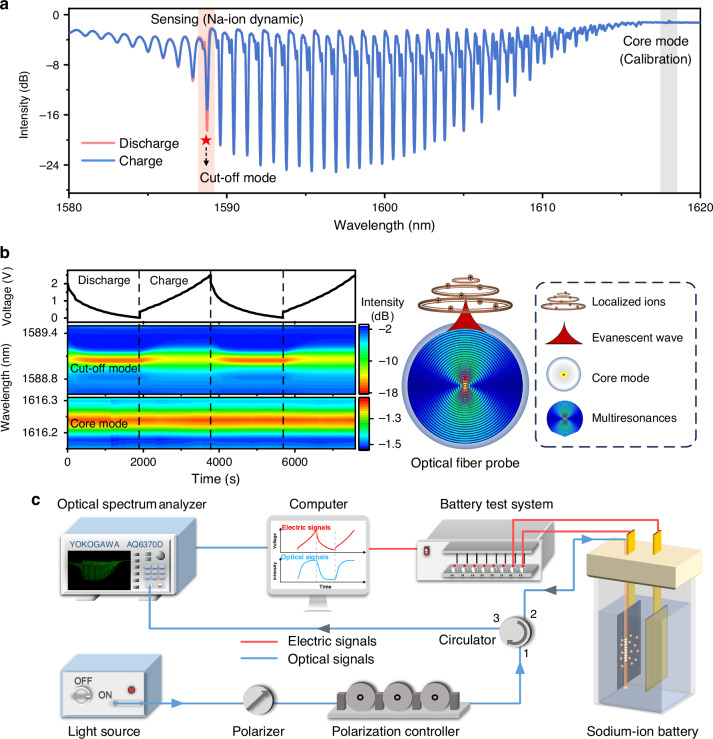
**Spectral response characteristics of the MFG sensor and integrated optical and electrochemical sensing system for operando monitoring of NIBs.** **a** Reflection spectrum of the MFG sensor located at the electrode interface during battery charging and discharging. The cut-off mode (at the wavelength of 1589 nm) is used for high-sensitivity detection of ion concentration, whilst the core mode at 1616 nm is used to track and compensate for temperature fluctuations. **b** Real-time optical response during two charge/discharge cycles of an NIB. The diagram on the right shows the electric field intensity of modes across the fiber. **c** Experimental setup of an optical fiber sensing system for monitoring ion transport at the electrolyte-electrode interface in SIBs

In SIBs, typical comb-shaped reflectance spectra of the MFG sensor during charging and discharging are depicted in Fig. 5a. The MFG spectra include dozens of narrow cladding modes, ghost modes, and one core mode. These narrow cladding modes have a half-width of only 0.2 nm, rendering them highly sensitive to changes in the refractive index of the surrounding medium of the optical fiber. From a sensing mechanism perspective^46^, when the refractive index of the external medium exceeds the effective refractive index of a specific cladding mode, the energy of that cladding mode leaks from the fiber cladding into the external environment. This leakage forms a leaky mode whose mode field is perpendicular to the fiber and the external tangent plane, and it propagates sinusoidally. On the other hand, when the refractive index of the external medium is lower than the effective refractive index of a certain cladding mode, the energy of that cladding mode is predominantly trapped in the fiber cladding, constituting a guided mode. Regarding the boundary condition between leaky and guided cladding modes, a cut-off mode is defined when the effective refractive index of a specific high-order cladding mode equals the refractive index of the external medium. This cut-off mode is used for precise calibration of the external refractive index (a comprehensive analysis has been presented in Fig. S12)^47^. The cladding mode labeled with a red star on the left side of Fig. 5a corresponds to the cut-off mode in the electrolyte of the SIB, exhibiting high sensitivity to variations in ion concentration at the electrode-electrolyte interface. The MFG sensor operates in the near-infrared range, where the evanescent field decay scales proportionally with wavelength. This configuration achieves an extended evanescent penetration depth (~1.5 μm), enabling effective coverage of the most active region for ion transport and electron transfer at the electrode surface. Simultaneously, the reflection peak at 1618 nm marked with gray shading on the right side of Fig. 5a represents the core mode. Due to the confinement of the core mode’s propagation within the core and its independence of the surrounding medium, it proves useful for temperature measurement in this experiment.

Figure 5b illustrates GCD curves (top) during two cycles for Na-ion storage at 400 mA g^-1^, along with real-time spectral responses of the MFG cut-off mode and core mode (shown in the middle and bottom traces, respectively). We present contour plots to visually represent changes in resonance mode intensity and wavelength, with color shifts indicating intensity fluctuations. The intensity of the cut-off mode (Fig. 5b, middle) gradually increases during the discharge process, reaching its maximum at the end of discharge, and rapidly decreases during the current reversal (charging), accompanied by an increase in bandwidth. The second cycle demonstrates good reproducibility. Concentration calibration results of the electrolyte indicate that the increase in optical intensity confirms the increase in Na-ion concentration (see complete analysis in Fig. S6). Therefore, the increased optical intensity during the discharge process corresponds to an increase in Na-ion concentration at the interface position, suggesting electrochemical reactions at the electrode with Na-ion. Both the spectral mapping (Fig. 5b, bottom) and quantitative tracking (Fig. S13) show that the core mode exhibited negligible fluctuations in both resonance wavelength and peak intensity during cycling, in sharp contrast to the dynamic response of the cut-off mode. This invariance confirms that the MFG sensor experienced no significant mechanical strain or bending artifacts, and that temperature effects on the experimental results can be excluded.

If the core mode does change (in intensity or wavelength), we can calibrate the spectral ion concentration sensing relative to the core mode to adapt to local temperature changes. This is because both modes exhibit the same temperature sensitivity, as shown in Fig. S14. Therefore, in practical applications, the MFG sensor can not only monitor real-time changes in ion concentration inside the battery but also temperature, effectively decoding the influence of temperature variations on ion concentration sensing.

### Sensing system

The operando sensing system for monitoring the ion transport dynamics during the SIB electrochemical process consists of two major subsystems (Fig. 5c): an optical system used for in situ detection and an electric system used for driving and calibration. Both optical and electrical signals are recorded and analyzed in real-time by a computer. The sensing system includes a 1520–1620 nm broadband light source, a polarizer, a polarization controller, a circulator, an MFG sensing probe, and a spectrum analyzer. An electrochemical data acquisition system is used to collect electrochemical data, which is correlated with the optical signal. The complete optical fiber sensing probe is 10 mm long and has a diameter of 125 μm. To fabricate a practical working electrode, we cut a 1 × 2 cm^2^ piece of the electrode material and attached it to copper foil using sodium carboxymethylcellulose adhesive. An experimental battery with MFG was prepared in a sealed quartz electrolysis cell with a metallic sodium counter electrode. 2 mL of electrolyte was injected into the battery to immerse the electrodes. The electrolyte we chose was 1 M NaClO_4_ dissolved in diethyl carbonate/ethylene carbonate (1:1 vol%) with 5 wt% fluoroethylene carbonates. The optical fiber sensing probe was carefully attached to the working electrode. The top of the cell was sealed with paraffin wax to maintain its airtightness while allowing optical and electrical connections to pass through. Finally, the measurement setup shown in Fig. 5c can be made considerably more compact and inexpensive by replacing the broadband source and optical spectrum analyzer with a tunable or fixed laser diode and a power meter^45^, respectively, as shown in Fig. S15.

## Supplementary information

Supplementary information for *Operando* tracking of ion kinetics and state-of-charge via multiresonant fiber-optic grating sensors in sodium-ion batteries

## Data Availability

The authors declare that the main data supporting the findings of this study are available within the article and its Supplementary Information files. All other relevant data supporting the findings of this study are available from the corresponding author on request.
