# Supplementary material for: Operando tracking of ion kinetics and state-of-charge via multiresonant fiber-optic grating sensors in sodium-ion batteries
Source: Light Sci Appl. 2026 Jun 26;15:288. doi: 10.1038/s41377-026-02388-1 (PMC13309571; doi:10.1038/s41377-026-02388-1)
Supplement: Supplementary file 1 — Supplementary information for Operando tracking of ion kinetics and state-of-charge via multiresonant fiber-optic grating sensors in sodium-ion batteries [file 41377_2026_2388_MOESM1_ESM.docx]

**Supplementary information**

***Operando* tracking of ion kinetics and state-of-charge via multiresonant fiber-optic grating sensors in sodium-ion batteries**

Xile Han^1,a^, Jinliang Li^1,a^, Wen Wu^1,a^, Jiajian Long^a^, Xumiao Chen^c^, Yongqi Li^d^, Man Chen^d^, Gaozhi Xiao^e^, Yongjin Fang^c,^*, Wenjie Mai^b,^*, and Tuan Guo^a,^*

^a^Institute of Photonics Technology, College of Physics & Optoelectronic Engineering, Jinan University, Guangzhou, Guangdong 510632, China

^b^Department of Physics, College of Physics & Optoelectronic Engineering, Jinan University, Guangzhou 510632, China

^c^College of Chemistry and Molecular Sciences, Hubei Key Laboratory of Electrochemical Power Sources, Wuhan University, Wuhan 430072, China

^d^China Southern Power Grid Power Generation Company Limited, Energy Storage Research Institute, Guangzhou 510080, China

^e^Advanced Electronics and Photonics Research Centre, National Research Council of Canada, Ottawa K1A 0R6, Canada

^1^These authors contributed equally.

*Corresponding authors’ email: Y. Fang (fangyj@whu.edu.cn), W. Mai (wenjiemai@email.jnu.edu.cn), T. Guo (tuanguo@jnu.edu.cn)

**Fabrication of multiresonant fiber grating (MFG) sensor**

An 18 mm MFG is embedded in a single-mode photosensitive optical fiber (FIBERCORE PS1250/1500) with a diameter of 125 μm. Using a ArF excimer laser emitting pulses at a wavelength of 193 nm and a phase mask with a period of 1117 nm, periodic interference fringes are generated in the fiber core, achieving permanent refractive index modulation. The synthesized MFG exhibits a core mode Bragg resonance at 1616 nm and several cladding mode resonances within the 1550-1620 nm spectral range. The tilt angle determines the wavelength spacing between high-order cladding modes and the Bragg mode. In an electrolyte solution with a refractive index of 1.39 to 1.44, a tilt angle of 10° was chosen to maximize the resonance intensity of the interface mode (measured using a digital refractometer, Reichert 13940000). Finally, to obtain the reflection spectrum, the fiber was cut a few millimeters downstream of the grating and the end face was coated with a gold layer to increase broadband reflectivity. Another significant advantage of the reflection structure is that it avoids crosstalk in the evanescent field of the MFG, as there is no need to apply tension to keep the fiber straight. It is important to note that the operando measurements were performed in an open-cell configuration (as shown in Figure S1(a)), which allows for free volume expansion of the electrode, thereby preventing the accumulation of mechanical pressure typical of confined coin cells.

**Figure S1 Photographs of optical fiber sensing at the electrode-electrolyte interface.** (a, b) Photographs of the configuration for the NIB and electrode-electrolyte interface. The inset in (a) shows a dark field optical microscope image and a cross-sectional view of the optical fiber and electrode: the MFG probe is attached to surface of the working electrode. (c) Photograph of the optical fiber sensor. (d) Structural schematic diagram of the MFG.

**SnO_2_/BaTiO_3_/C nanofibers preparation**

The free-standing SnO_2_/BaTiO_3_/C nanofibers were prepared by electrospinning. SnO_2_ and BaTiO_3_ nanoparticles were ground together in a 4:1 mass ratio for 2 h. Polyacrylonitrile (1.0 g) and Pluronic F127 (25 mg) were dissolved in dimethylformamide (10 mL) and stirred for 12 h to obtain a homogeneous solution. The ground SnO_2_/BaTiO_3_ composite (1.12 g) was then added to the solution, followed by ultrasonic treatment and stirring for another 12 h. The resulting solution was loaded into a 10 mL syringe pump and underwent an electrospinning process at a feed rate of 1 mL h^-1^ and a collection distance of 12 cm, under an applied voltage of 16.5 kV. Additionally, a dehumidifier was used to maintain the room humidity at approximately 40% to ensure the stability of the nanofibers during the process. The collected film was dried at 60 °C overnight, then heat-treated at 230 °C in air and 500 °C in argon (2 h each), achieving carbonization and enhancing conductivity.

**Cell assembly and electrochemical test**

To evaluate the electrochemical performance of the electrode material, we cut the free-standing electrode material into discs with a diameter of 14 mm as the work electrode. The batteries were packaged in a glove box (Etelux-Lab2000) filled with argon gas (O_2_ < 0.1 ppm, H_2_O < 0.1 ppm). Metallic Na foil was used as the counter electrode. 1 M NaClO_4_ dissolved into ethylene carbonate (EC)/diethyl carbonate (DEC) mixed solution (1:1 w/w) with 5 wt% fluoroethylene carbonate (FEC) was used as an electrolyte, and Whatman glass fiber was applied to the separator. The constant current charge-discharge curves and cycling performance of the batteries were recorded using a battery testing system (LAND CT2001A). Cyclic voltammetry (CV) was measured on an electrochemical workstation (ChenHua, CHI 1030C) at a scan rate of 0.1 mV s^-1^ unless otherwise stated.

**Characterization**

Scanning electron microscopy (SEM) measurements were performed using a Hitachi S-4800 field emission scanning electron microscope equipped with an energy dispersive X-ray spectrometer (EDS). X-ray photoelectron spectroscopy (XPS) measurements were performed using a Phi X-tool XPS instrument. The crystal structures of the materials were confirmed by X-ray diffraction (XRD) analysis using a Rigaku diffractometer.


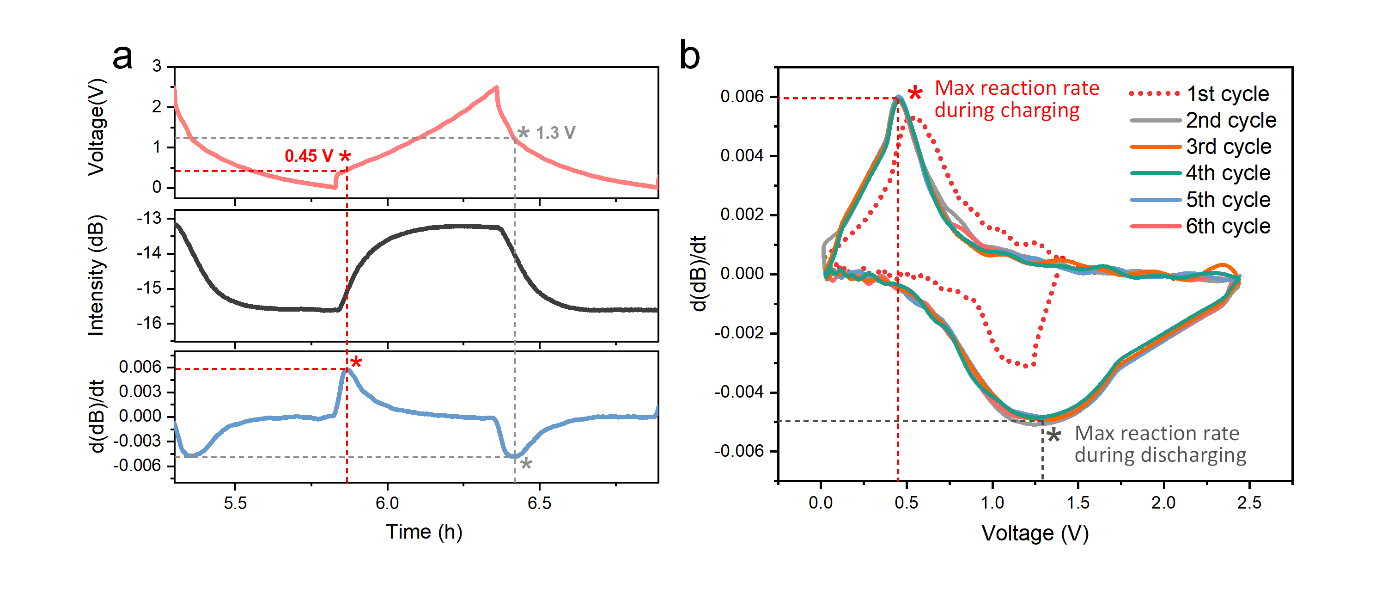


**Figure S2 Correlation between the optical derivative d(dB)/dt and the voltage.** (a) Real time response of GCD curve (red), optical intensity (black), and optical derivative (blue) at 400 mA·g^-1^. The red and gray asterisks mark the peak reaction rates (maximum ion flux) during the charging and discharging phases, respectively, determined by the local extrema of the derivative curve. (b) The relationship curve between the optical derivative and the voltage at 400 mA·g^-1^. The marked peaks correspond to the potentials of maximum ion flux associated with the dominant redox reactions.


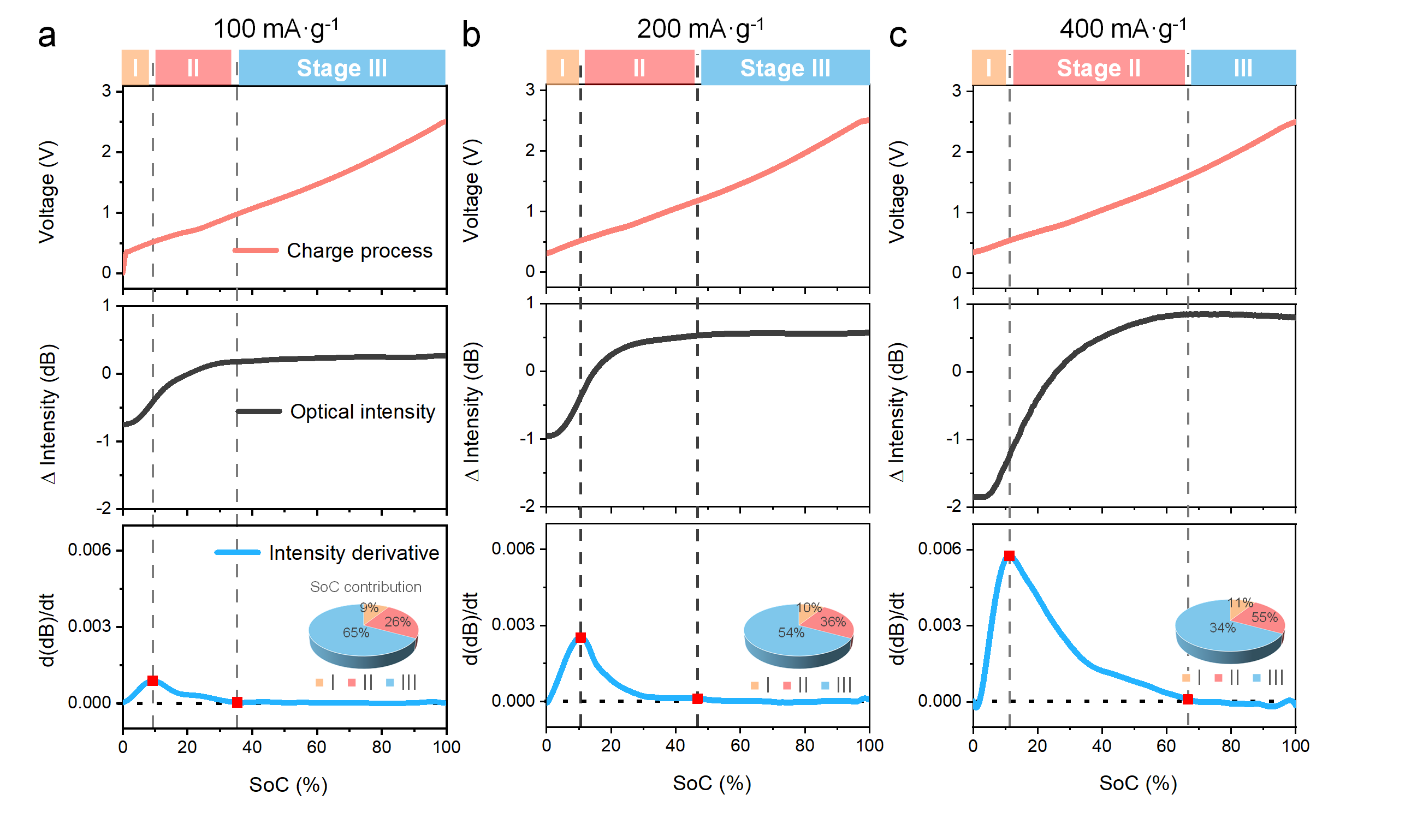


**Figure S3** **Operando decoding of dynamic ion processes of SnO_2_/BaTiO_3_/C nanofibers electrode during charging process.** Real time response of charge voltage (red), optical intensity (black), and optical derivative of NIBs at (a) 100 mA·g^-1^, (b) 200 mA·g^-1^ and (c) 400 mA·g^-1^. The chart in the third row gives the state of charge (SoC) contribution proportion for each stage.


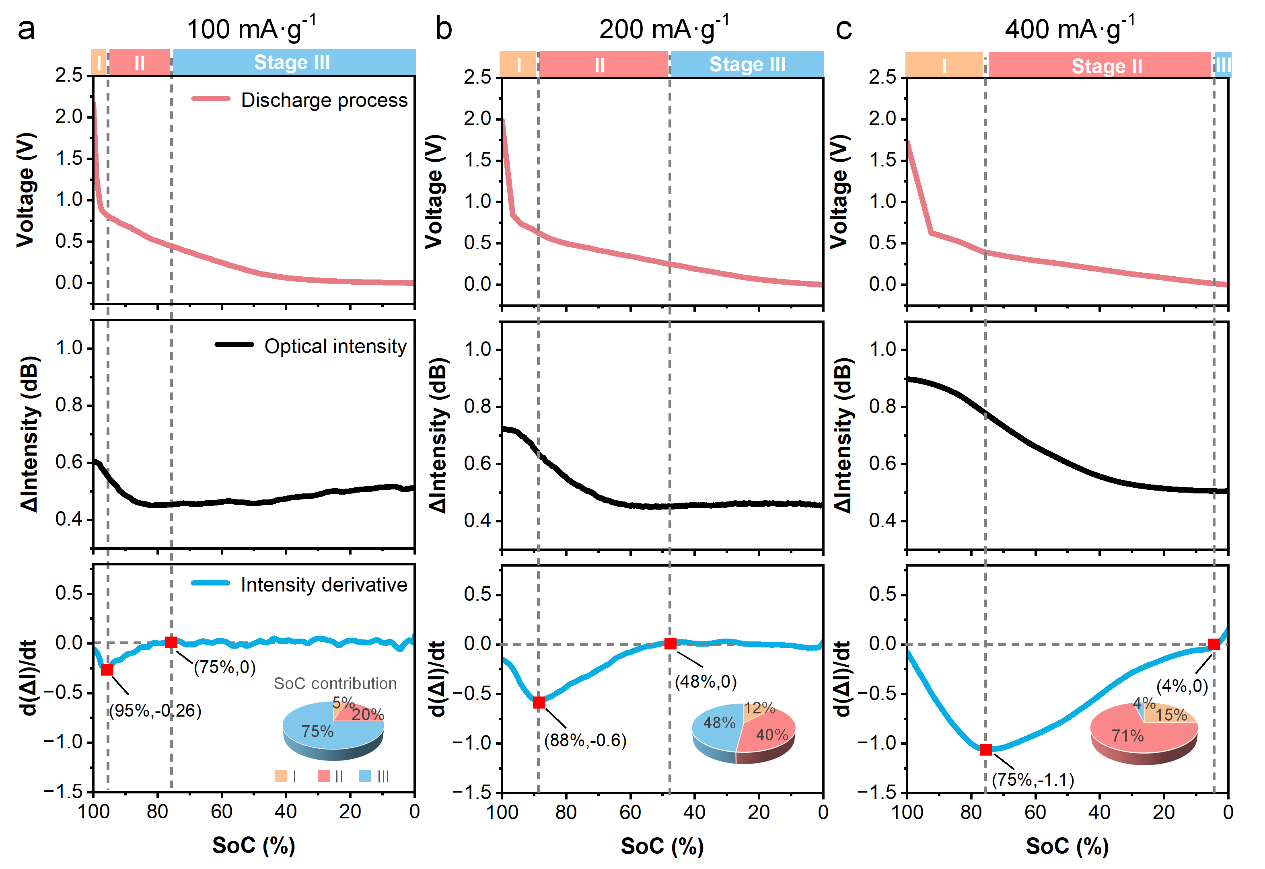


**Figure S4 Operando decoding of dynamic ion processes of hard carbon electrode during discharging process.** Real time response of charge voltage (red), optical intensity (black), and optical derivative of NIBs at (a) 100 mA·g^-1^, (b) 200 mA·g^-1^ and (c) 400 mA·g^-1^. The chart in the third row gives the state of charge (SoC) contribution proportion for each stage.


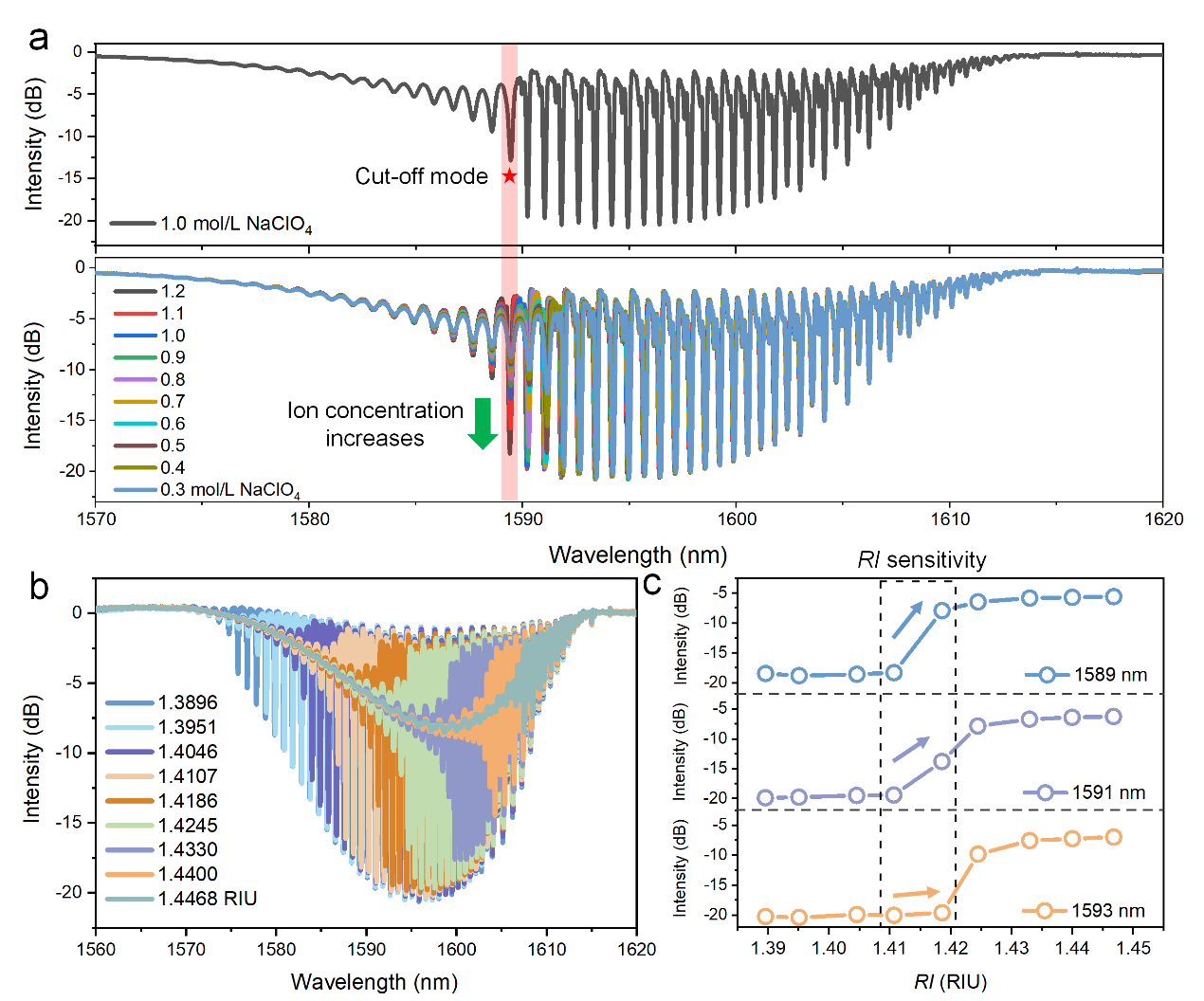


**Figure S5 Ion concentration and refractive index response of MFG spectra.** (a) The spectral response of a MFG with a tilt angle of 10° in NaClO_4_ EC:DEC:FEC with concentrations ranging from 0.3 to 1.2 M. The top graph shows the individual spectrum in the 1.0 M NaClO_4_ electrolyte, where the sensing mode is marked by a red asterisk; The wavelength of the sensing mode varies with the electrolyte. As the electrolyte concentration increases, the sensing mode shifts toward longer wavelengths, and its intensity increases. (b) The spectral responses of a MFG with a tilt angle of 10° in glycerol solution with a refractive index range of 1.3896-1.4468. The wavelength of the cut-off mode of MFG spectrum increases with increasing refractive index. (c) Intensity of the cut-off modes at the wavelengths of 1589 nm, 1591 nm, and 1593 nm as a function of the refractive index. The dashed rectangle identifies the *RI* sensitivity of the three modes within the useful measurement range of the studied example (approximately 1.415 RIU). The mode at the wavelength of 1589 nm demonstrates maximum *RI* sensitivity.


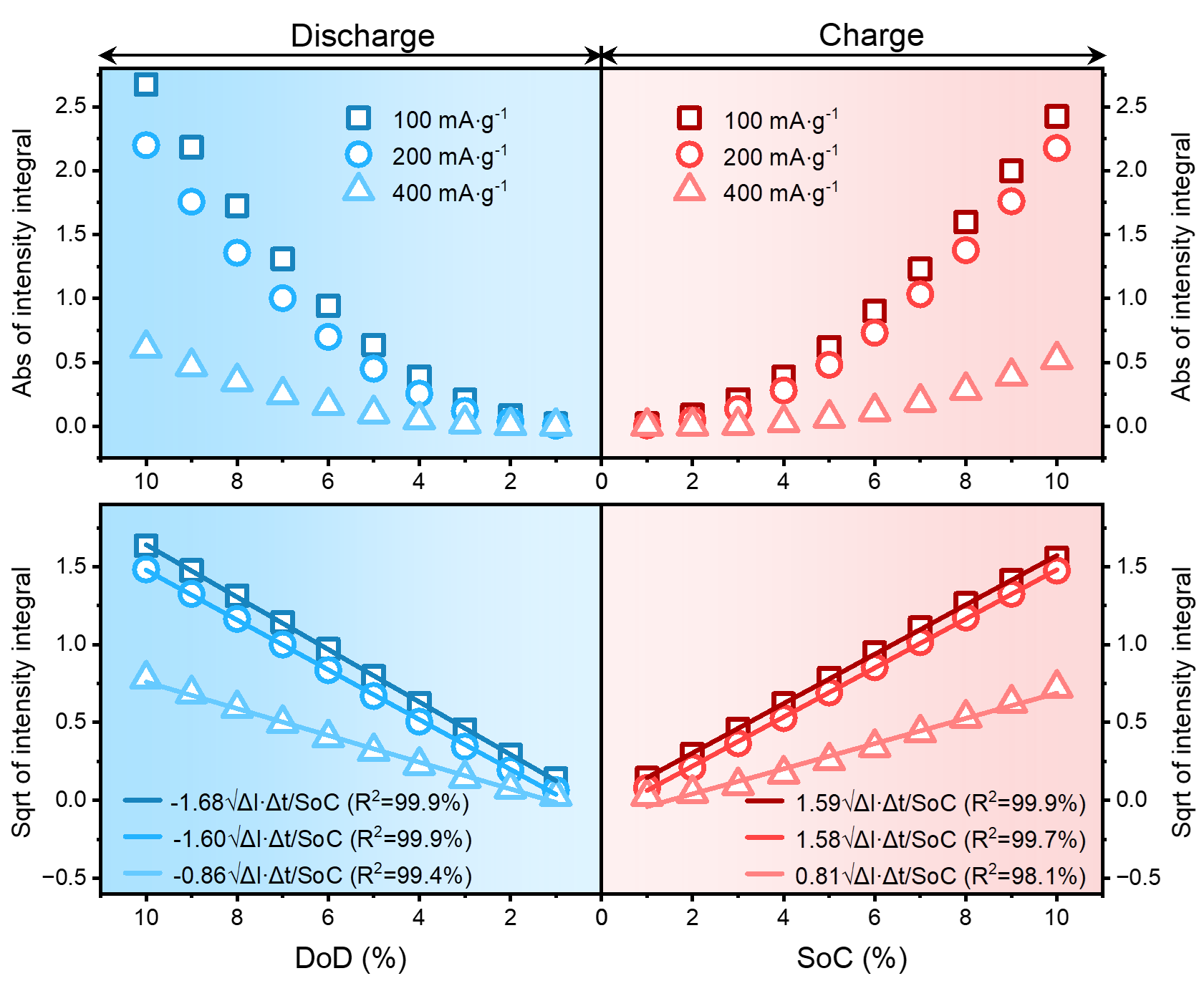


**Figure S6** The relationships between the SoC changes and the integral of the change in optical intensity in discharge (blue) and charge (red) processes in initial 10% SoC state (top), and we found that the relationship between SoC changes and the integral of the change in optical intensity present nonlinear relationship at this stage. The relationships between the SoC changes and the sqrt integral of the change in optical intensity in discharge (blue) and charge (red) processes in initial 10% SoC state (bottom).


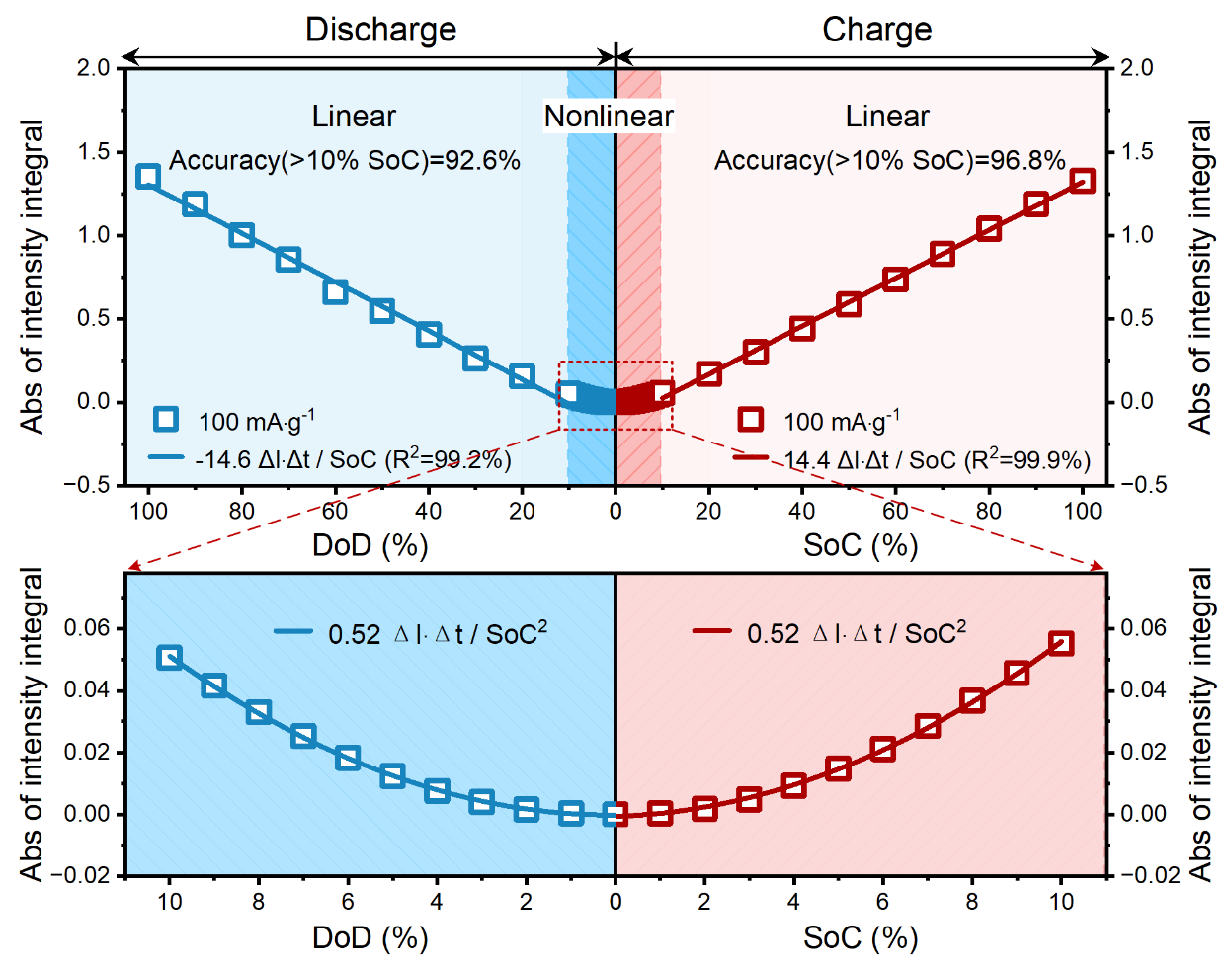


**Figure** **S7** Relationships between the integral of the change in optical intensity and the SoC during discharging (blue) and charging (red) at 100 mA g^-1^ in hard carbon. According to the calculations, in the linear region, the linearity between the SoC and the integral of the change in optical intensity is 99.2% (discharging) and 99.9% (charging), with the accuracy of 92.6% (discharging) and 96.8% (charging). In the enlarged nonlinear region, the binomial relationship between the integral of the optical intensity change and the SoC is still satisfied.


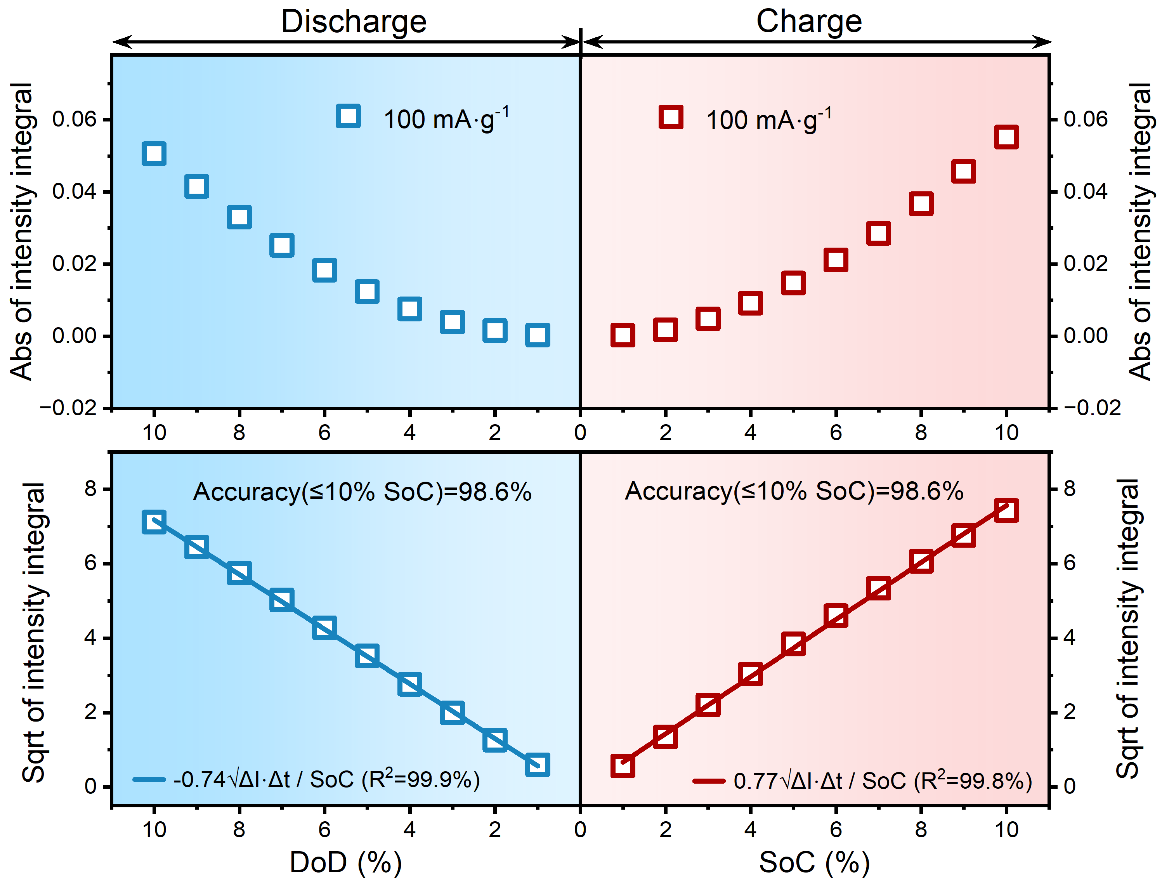


**Figure S8** Relationships between the integral of the change in optical intensity and the SoC during discharging (blue) and charging (red) at 100 mA g^-1^ in hard carbon. According to the calculations, in the non-linear region, the linearity between the SoC and the sqrt integral of the change in optical intensity is 99.9% (discharging) and 99.8% (charging), with the accuracy of 98.6% (discharging) and 96.7% (charging).

**
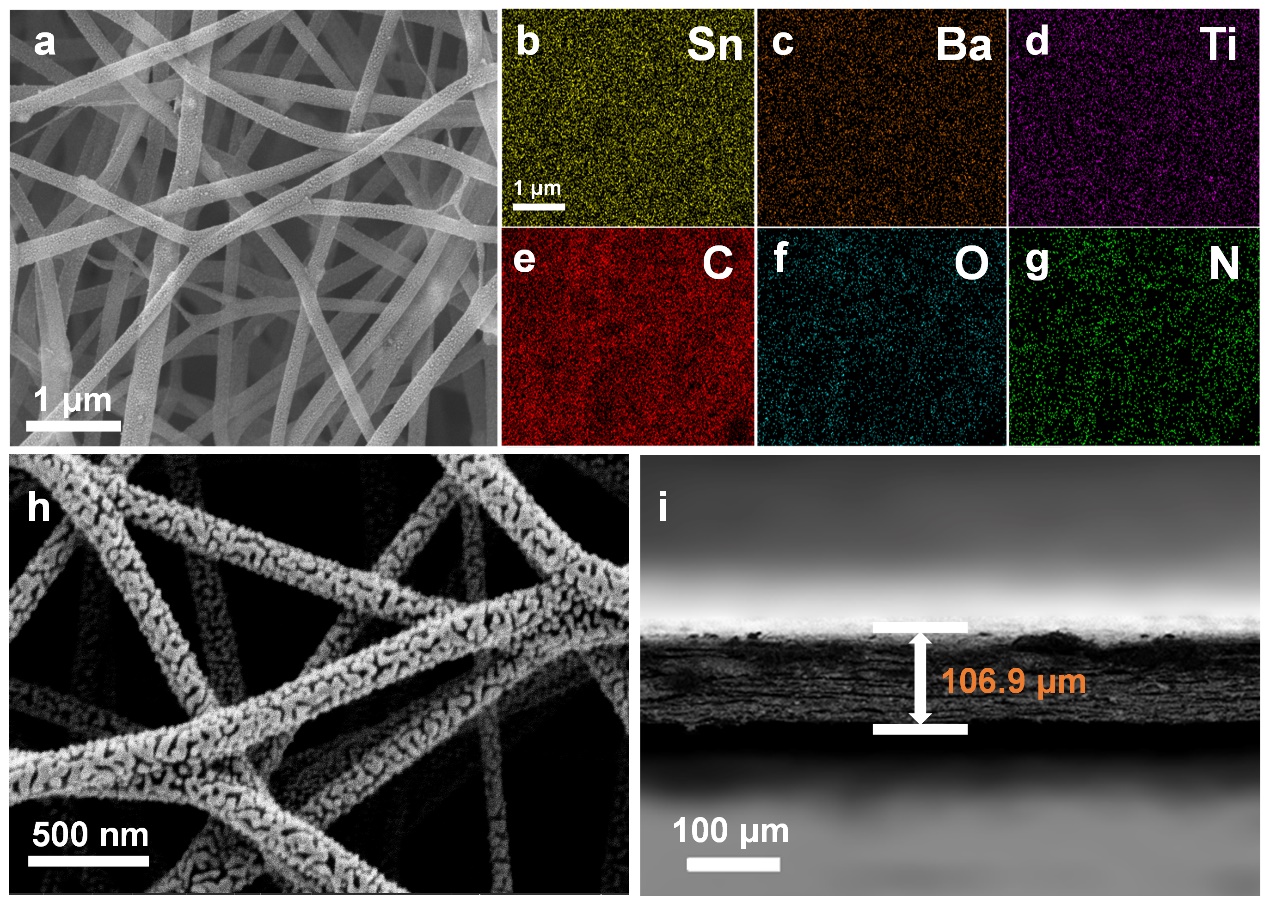
**

**Figure S9** (a) SEM image of SnO_2_/BaTiO_3_/C nanofibers. EDS element mapping of (b) Sn, (c) Ba, (d) Ti, (e) C, (f) O, (g) N in SnO_2_/BaTiO_3_/C nanofibers. (h) The SEM images at the surface of fresh SnO_2_/BaTiO_3_/C nanofibers. (i) The cross-sectional thickness of the SnO_2_/BaTiO_3_/C nanofibers.


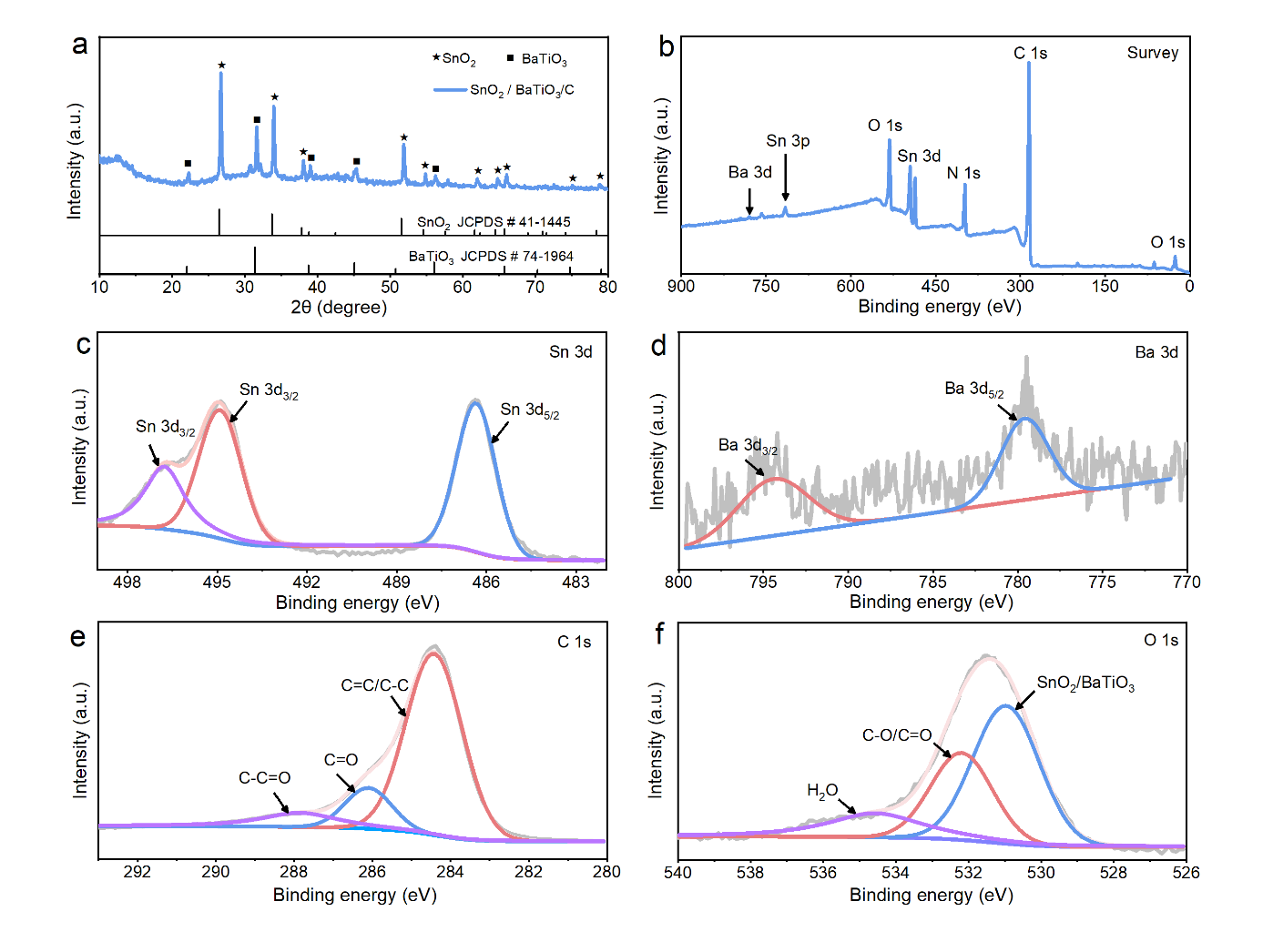


**Figure S10 Material structure and interface characterization.** (a) XRD, (b) survey, (c) Sn 3d, (d) Ba 3d, (e) C 1s and (f) O 1s XPS of SnO_2_/BaTiO_3_/C nanofibers.


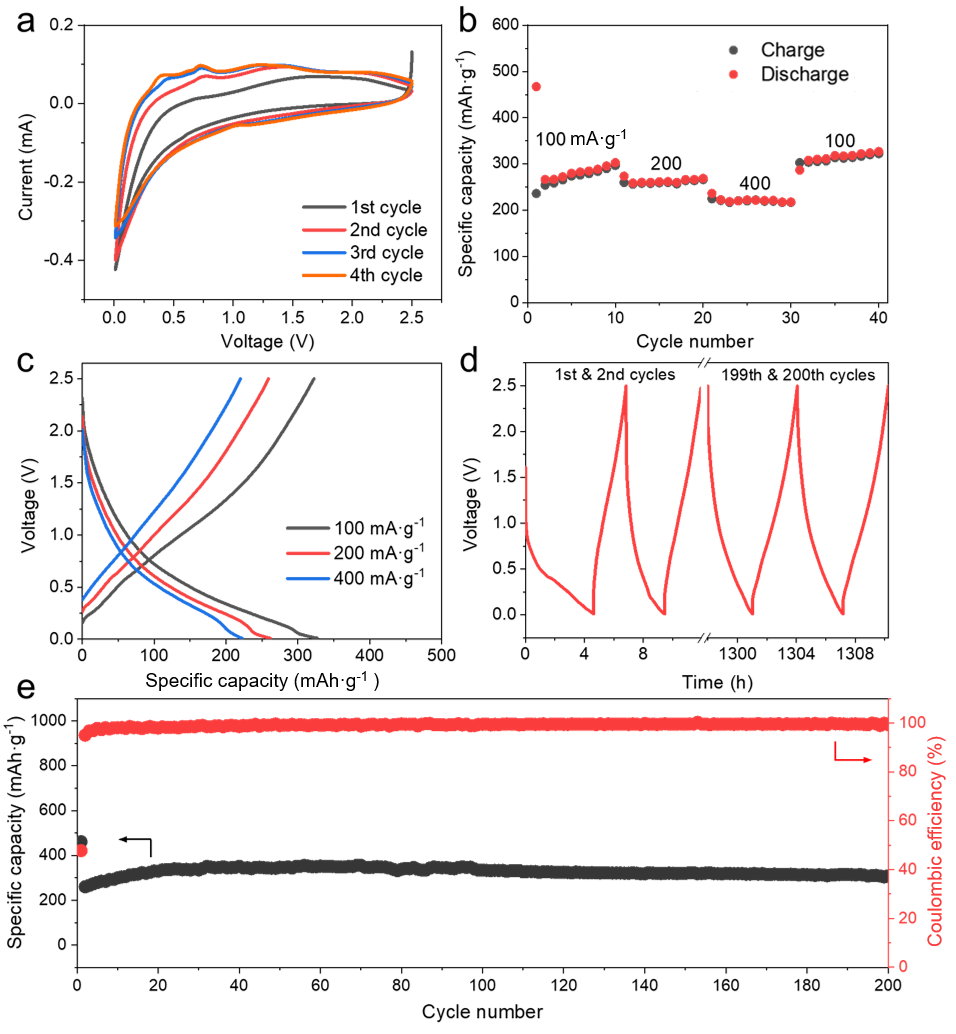


**Figure S11 Cycling performance of SnO_2_/BaTiO_3_/C nanofibers.** (a) Cyclic voltammetry curves of SnO_2_/BaTiO_3_/C nanofibers at 0.1 mV·s^-1^. (b) Rate performance and (c) GCD profiles of SnO_2_/BaTiO_3_/C nanofibers at different current density. (d) The voltage-time profile of the first two and last two cycles of the battery. (e) Long cycling stability of SnO_2_/BaTiO_3_/C nanofibers at 100 mA·g^-1^.


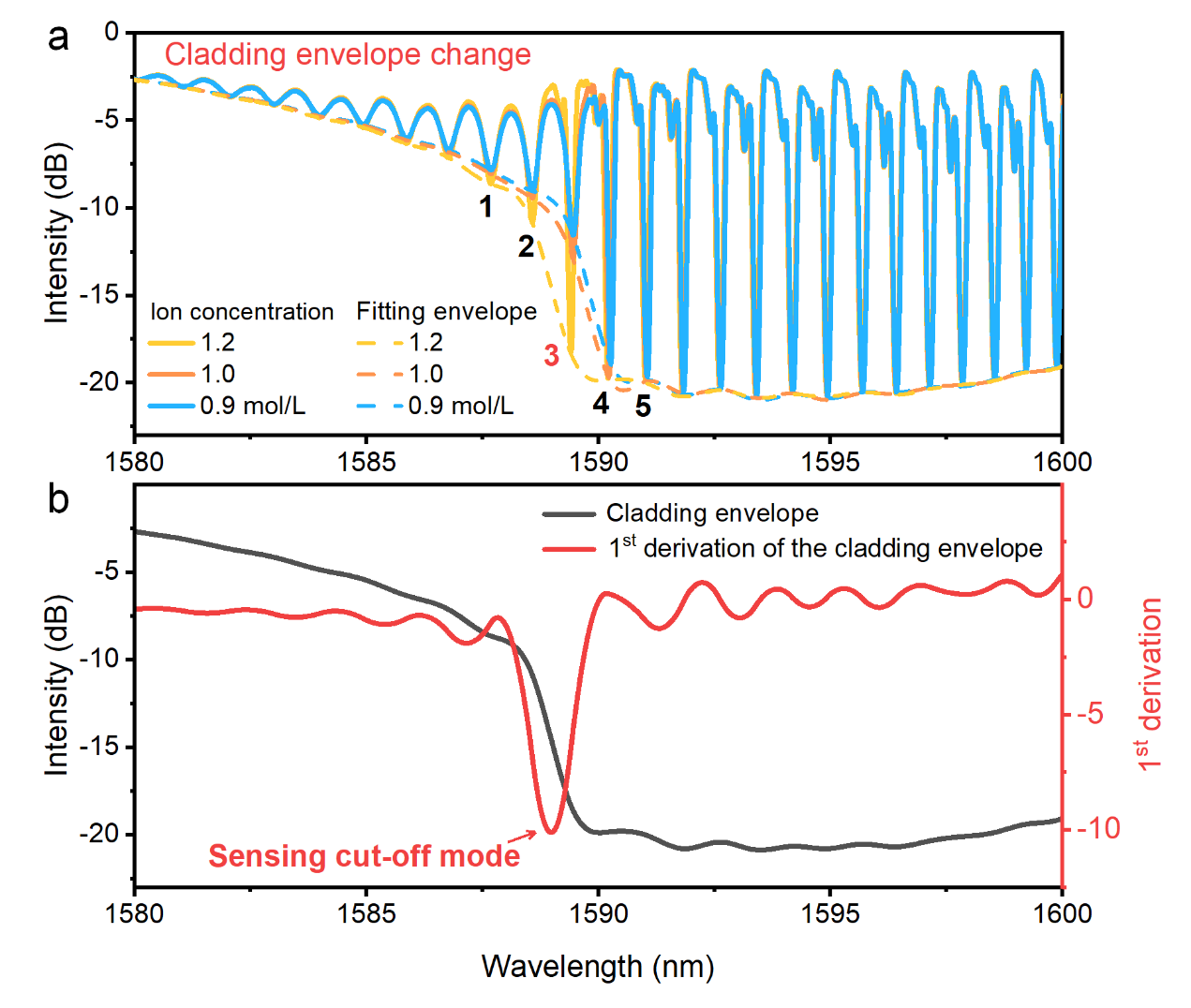


**Figure S12** **The principle of selection of the most sensitive interface mode.** (a) The measurement of a 10° MFG as a function of external refractive index (solid line), where all attenuated envelope modes within the attenuation region are labeled from 1 to 5. The fitted envelope of the attenuation cladding mode resonance shifts toward lower intensity (dashed line). The cladding mode labeled as 3 exhibits the steepest slope, indicating the highest sensitivity to RI variations, particularly to subtle surface RI perturbations occurring during the charging/discharging process inside the batteries.


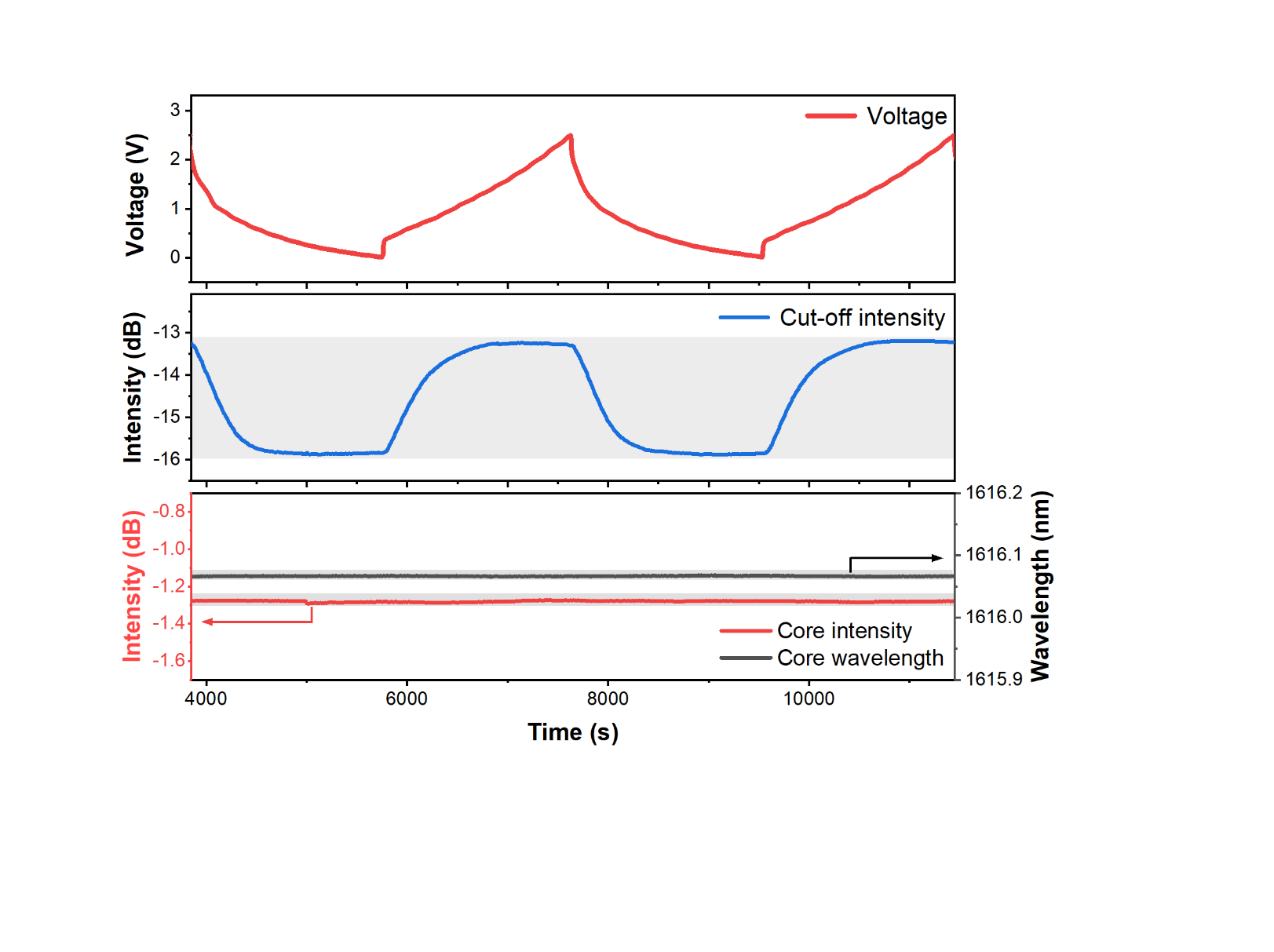


**Figure S13** Real-time optical response during two charge-discharge cycles in NIBs.


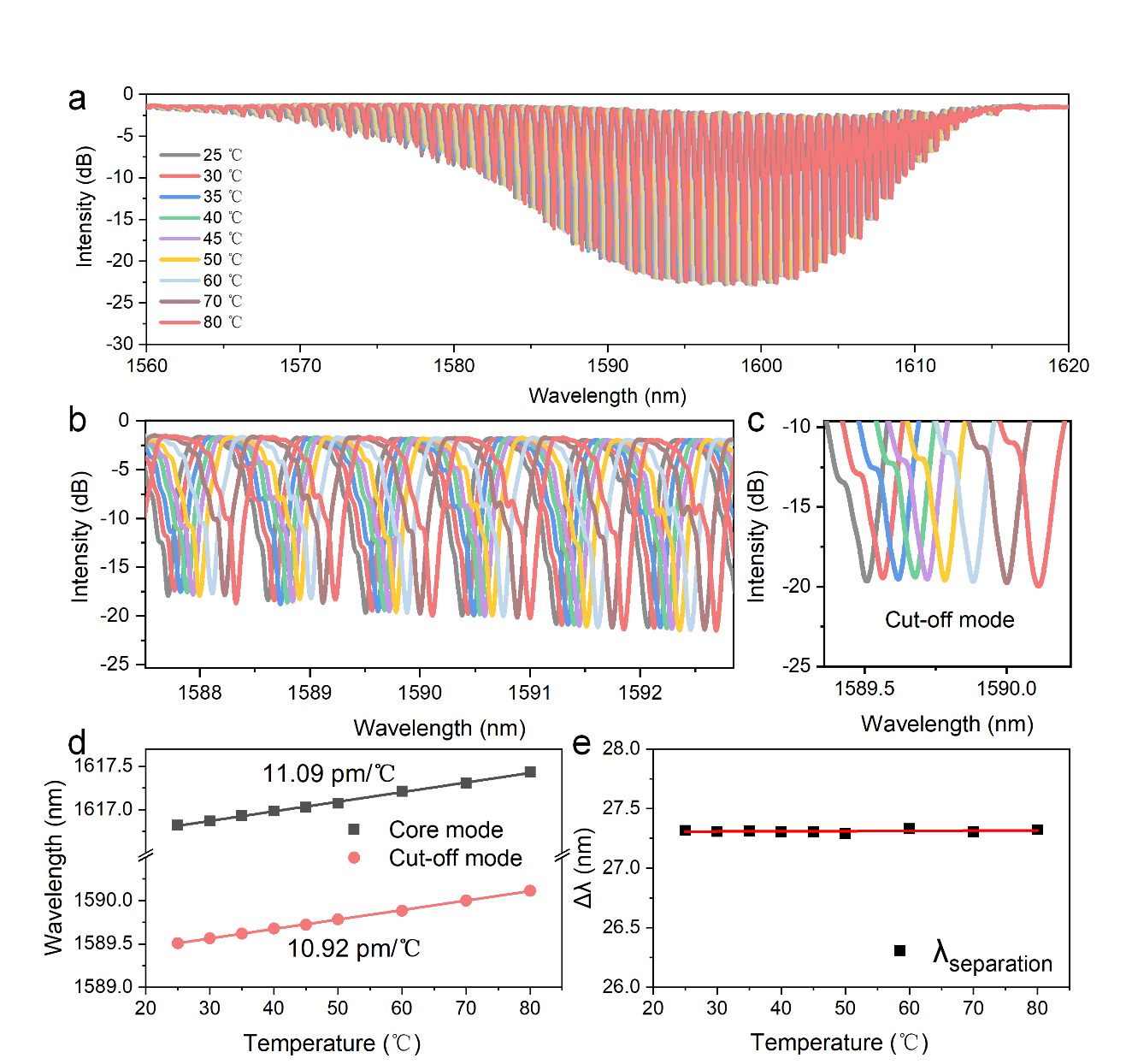


**Figure S14 Response of the MFG spectrum to temperature.** (a) The full spectral response of a MFG at 25 to 80 °C in air. (b, c) Enlarged spectrum of the claddings at ~1590 nm and cut-off mode. The arrows indicate the direction of wavelength change during increase of temperature. (d) Linear relationship between temperature change and core mode wavelength shift and cut-off mode wavelength shift. Both modes have very close temperature sensitivities. (e) Δλ between the core mode and the cut-off mode as a function of temperature. Δλ is not affected by temperature. Temperature crosstalk can be eliminated through Δλ decoupling. Based on these results, we can calibrate the spectral ion concentration sensing relative to the core mode to accommodate local temperature variations. This is because both modes exhibit the same temperature sensitivity. Consequently, in practical applications, the MFG sensor can not only monitor real-time changes in ion concentration within the battery but also detect internal temperature. This dual functionality effectively eliminates the impact of temperature fluctuations on the ion concentration sensing.


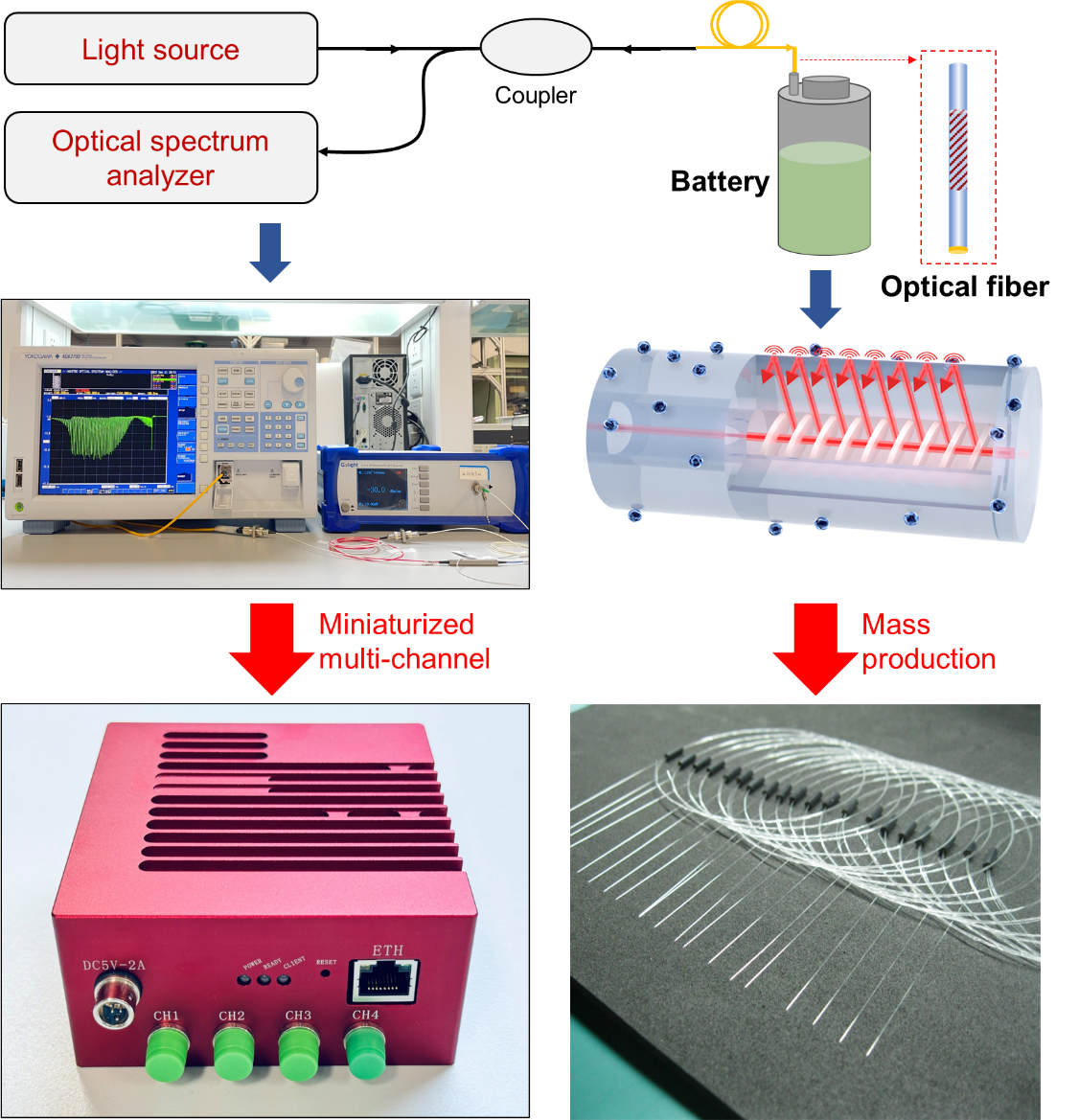


**Figure S15** The portable fiber grating interrogator for in-field measurement. In this setup, a tunable laser (TLS) replaces the broadband light source as the light source, accompanied by a photodiode serving as the photodetector and an analog-to-digital (A/D) converter for data acquisition, thus replacing the optical spectrum analyzer. The TLS is utilized to align with the wavelengths corresponding to the most sensitive multiresonant coupling modes. Once the sensor is characterized, it can be replaced with a common laser, such as a compact VCSEL. This technique relies on edge filtering, where the optical power change is induced by the wavelength shift of the mode concerning the fixed wavelength of the laser source. Our laboratory has developed a prototype of this instrumentation utilizing the aforementioned power detection principle.
